# Single-cell recordings in the human medial temporal lobe

**DOI:** 10.1111/joa.12228

**Published:** 2014-08-28

**Authors:** Hernan G Rey, Matias J Ison, Carlos Pedreira, Antonio Valentin, Gonzalo Alarcon, Richard Selway, Mark P Richardson, Rodrigo Quian Quiroga

**Affiliations:** 1 Centre for Systems Neuroscience, University of LeicesterLeicester, UK; 2 Department of Engineering, University of LeicesterLeicester, UK; 3 Department of Experimental Psychology, University of OxfordOxford, UK; 4 Department of Clinical Neuroscience, King’s College LondonLondon, UK; 5 Department of Clinical Neurophysiology, King’s College HospitalLondon, UK; 6 Departamento de Fisiología, Universidad ComplutenseMadrid, Spain; 7 Department of Neurosurgery, King’s College HospitalLondon, UK; 8 Department of Neurology, King’s College HospitalLondon, UK

**Keywords:** declarative memory, medial temporal lobe, repetition suppression, single cell recordings in humans

## Abstract

Recordings from individual neurons in patients who are implanted with depth electrodes for clinical reasons have opened the possibility to narrow down the gap between neurophysiological studies in animals and non-invasive (e.g. functional magnetic resonance imaging, electroencephalogram, magnetoencephalography) investigations in humans. Here we provide a description of the main procedures for electrode implantation and recordings, the experimental paradigms used and the main steps for processing the data. We also present key characteristics of the so-called ‘concept cells’, neurons in the human medial temporal lobe with selective and invariant responses that represent the meaning of the stimulus, and discuss their proposed role in declarative memory. Finally, we present novel results dealing with the stability of the representation given by these neurons, by studying the effect of stimulus repetition in the strength of the responses. In particular, we show that, after an initial decay, the response strength reaches an asymptotic value after approximately 15 presentations that remains above baseline for the whole duration of the experiment.

## Introduction

It has long been established that the ventral visual pathway has an important role in visual perception and recognition (Logothetis & Sheinberg, 1996; Tanaka, 1996; Tsao & Livingstone, 2008). As we move forward along the pathway, we go from neurons in V1 responding to very low-level features of the image sensed by the retina (e.g. showing selectivity to local orientations; Hubel & Wiesel, 1962), to neurons in the infero-temporal cortex (ITC) responding to high-level features of the image (e.g. faces; Gross, 2008). From ITC there are direct projections to the medial temporal lobe (MTL; Saleem & Tanaka, 1996; Suzuki, 1996), a brain region that includes the hippocampus, the amygdala, and entorhinal, parahippocampal and perirhinal cortices, structures exhibiting complex connectivity patterns between them and with the neocortex (Suzuki, 1996; Burwell & Agster, 2008).

There is a large amount of evidence supporting a key role of the MTL in declarative memory, from studies in animals to evaluations of patients with lesions (Squire & Zola-Morgan, 1991; Eichenbaum, 2001; Squire et al. 2004; Moscovitch et al. 2005). In order to study such memory processes, human subjects appear to be the ideal model, given the large amount of rich experiences they can recall and report. However, the study of the human brain has been mainly carried out using non-invasive techniques, such as functional magnetic resonance imaging (fMRI) and (scalp) electroencephalogram (EEG). The main caveat of these techniques is that they give very limited and indirect information of the neural processes underlying brain functions. Ideally, one would like to study the activity of single cells directly, but single-cell studies in humans are very limited due to obvious ethical reasons. Nevertheless, patients suffering certain disorders, such as Parkinson’s disease, dystonia or epilepsy, might require invasive recordings in order to obtain an accurate diagnosis and/or appropriate treatment (Engel et al. 2005). This allows intracranial recordings in the MTL in humans, given that this area is involved in certain forms of epilepsy (Niedermeyer, 1993). Moreover, with the proper setup, it is possible to record not only intracranial EEG or local field potentials (LFPs) – which account for mean activation patterns across several neurons– but also the electrical activity of individual neurons (Quian Quiroga & Panzeri, 2009).

In the late 1970s, the first studies on single-cell activity in the human MTL during memory tasks took place (Halgren et al. 1978). In the following years, several experiments to study different aspects of human cognition – including language, memory, perception, emotion – were developed (Fried et al. 1997; Ojemann et al. 1988, 1998; see Mukamel & Fried, 2012 for a review) and in 2000, the presence of category-specific neurons (some responding only to faces, others to objects, others to animals, etc.) in the MTL was reported (Kreiman et al. 2000a). Following these findings, neurons in the MTL with highly selective and invariant responses were found (Quian Quiroga et al. 2005), and it was argued that these so-called ‘concept cells’ or ‘Jennifer Aniston neurons’ represent the meaning of the stimulus for declarative memory functions (Quian Quiroga et al. 2008a; Quian Quiroga, 2012a).

In this article we present different aspects related to the recording and behavior of ‘concept cells’ in the human MTL. We cover details of the surgical procedure and data acquisition, the experimental paradigms to study these neurons, the data processing necessary to analyze the neural responses, and discuss several properties of these cells that help understanding their role. We also present novel results dealing with the stability of the responses of concept cells, using a visual paradigm where each stimulus was shown between 20 and 30 times. This allowed us to study the modulation of the responses by stimulus repetition across several trials. We found that the strength of neuronal responses decreased until reaching a stable value above baseline firing levels.

## Surgical procedure and recordings

In about 25% of the patients suffering from epilepsy, antiepileptic drugs fail to control the seizures, what is known as refractory epilepsy (Schuele & Lüders, 2008; Kwan et al. 2011). Based on the results of several non-invasive tests (including scalp EEG, structural imaging and neuropsychological assessments) these patients might be candidates for a surgical solution, in which the epileptic focus is resected. However, before taking such a measure, it is clearly critical to have a reasonable degree of confidence about the location of the seizure focus. In some of these cases, non-invasive methods might not be sufficient to unambiguously localize the seizure onset zone, and intracranial recordings are required. This technique started in the 1930s and, at the time, intracranial recordings were performed only during surgery. Over the following decades, the technique for invasive recordings improved considerably (see Engel et al. 2005 for a timeline on the history of invasive recordings in the human brain). Nowadays, the subacute implantation of intracranial electrodes for an extended period allows continuous monitoring of the electrical activity in areas where the epileptic focus is presumed to be potentially located. Recordings are taken from several target areas and, in general, only a few of these sites end up being involved in the epileptogenesis (Mukamel & Fried, 2012). Depending on the rate of spontaneous seizures and the amount of data collected to localize the epileptic focus, patients might remain monitored from a few days to a couple of weeks.

The choice of the type of electrodes to be used depends on the location of the potential epileptic focus (or foci). A widely used type of intracranial electrodes is the subdural one, including strips and grids, consisting of an array of circular electrodes arranged along a line or a surface, respectively, and placed on top of the cortex. Grids usually require a full craniotomy for their implantation and they are particularly suitable for functional mapping of the cortex, as they provide a stable and dense array of recording electrodes. In areas responsible for language, motor or sensory function, the brain regions that must not be removed can be identified with electrical stimulation of the different grid contacts (Penfield, 1975). The advantage of strips is their larger flexibility for placement, for example along a cortical gyrus, and the fact that they can be inserted through a single burr hole using different trajectories.

**Figure 1:**
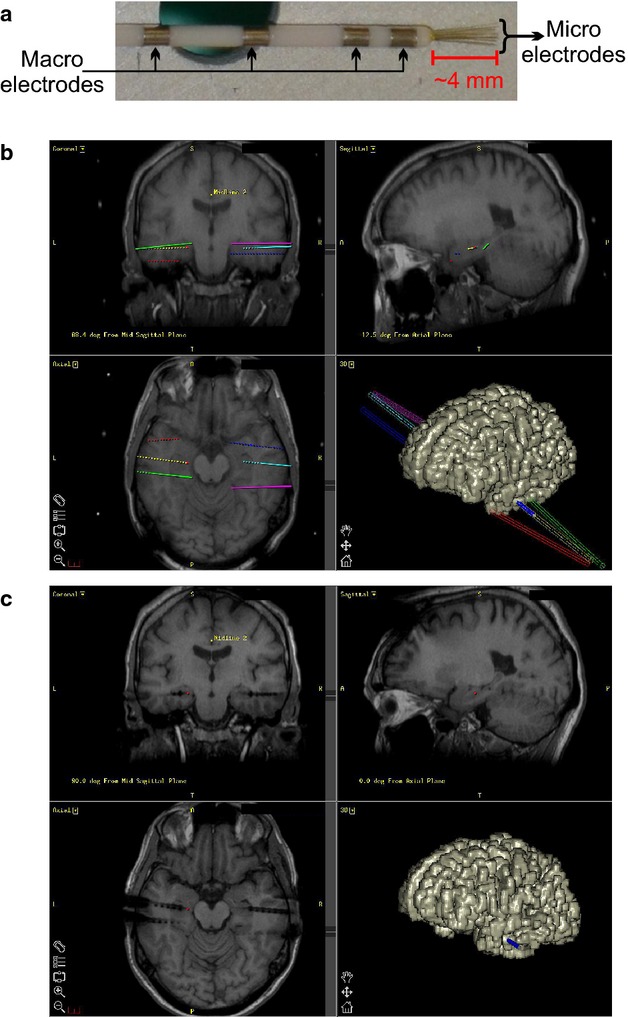
Surgical planning. (A) Before implantation, the microwires are inserted in the lumen of the polyurethane probe so they can be trimmed to the desired length (∼4 mm). (B) Based on a pre-operative MRI scan, computer software provides the stereotactic coordinates of the targets where the electrodes should be implanted. (C) After surgery, a post-operative MRI or CT is co-registered with the preoperative MRI to assess the accuracy of the actual position of the electrodes.

Nonetheless, in several cases the focus might be located in areas of the cortex that are difficult to be accessed with surface electrodes (orbitofrontal, cingulate, etc.) or subcortically, as in the case of the MTL. To target such areas the use of depth electrodes is often preferred. They are composed of a flexible polyurethane probe (Fig.1A) with low impedance platinum-iridium contacts that record intracranial EEG signals and, particularly, characteristic epileptogenic discharges or seizures. Their placement can be very accurate due to the use of stereotactic frames. In fact, preoperative MRI scans are typically used to establish the target position of the electrodes, based on clinical criteria, and the corresponding coordinates in the frame (Fig.1B). After surgery, a postoperative scan can be co-registered with the planning to verify the actual position of the electrodes (Fig.1C).

In the 1950s, the first single-units recordings from the human cortex were performed during surgery for epilepsy (Ward & Thomas, 1955). Single-cell recordings over extended periods of time were introduced in the early 1970s at the University California Los Angeles (UCLA) while patients remained in the hospital ward for seizure monitoring (Babb et al. 1973). These recordings were developed further at UCLA and nowadays they are performed by inserting platinum-iridium microwires (with a 40 μm width) inside the lumen of the probe containing the depth iEEG electrodes, which protrude 3–5 mm from the tip of the probe (Fig.1A; Fried et al. 1997). The microwires have high impedance (300–1000 kΩ) and are suitable for recording action potentials and LFPs.

The data we present in this study, both for illustrating single-cell responses and for studying the stability of these responses, were collected using commercially available (Ad-Tech) depth electrodes, where each bundle had eight active microwires and one low-impedance microwire that was used as local reference. All studies were performed at King’s College Hospital in London (UK) and were approved by its Research Ethics Committee. The electrodes were implanted bilaterally in the hippocampus and amygdala. Two patients were recorded using a 64-channel Digital Lynx system (Neuralynx), with the differential signal from each channel filtered between 0.1 and 9000 Hz, and sampled at 32 556 Hz. The other two patients were recorded using a 64-channel Neuroport system (Blackrock Microsystems), with the differential signal from each channel filtered between 0.3 and 7500 Hz, and sampled at 30 000 Hz. The data were processed offline, and the high-frequency activity (> 300 Hz) was extracted to identify the firing of the neurons (see Figs 3a and 4a for examples).

## Experimental paradigms

The prolonged nature of the monitoring of epileptic patients with intracranial recordings, in order to record a sufficient number of spontaneous seizures, provides a unique opportunity to record neural activity for several days while the patients perform different cognitive tasks. Experiments with conscious human subjects have several advantages over analogous experiments with animals as, in particular, humans can be easily instructed to perform complex tasks, thus avoiding overtraining. Moreover, they can deliver detailed feedback and they can be tested on their unique abilities, such as language (Ojemann et al. 1988) or verbal recall (Gelbard-Sagiv et al. 2008). Also, when studying human cognition, invasive recordings provide several advantages against non-invasive ones, such as an improvement in both temporal and spatial resolution, high signal to noise ratio and, particularly, the unique possibility of recording directly the activity of single neurons.

**Figure 2:**
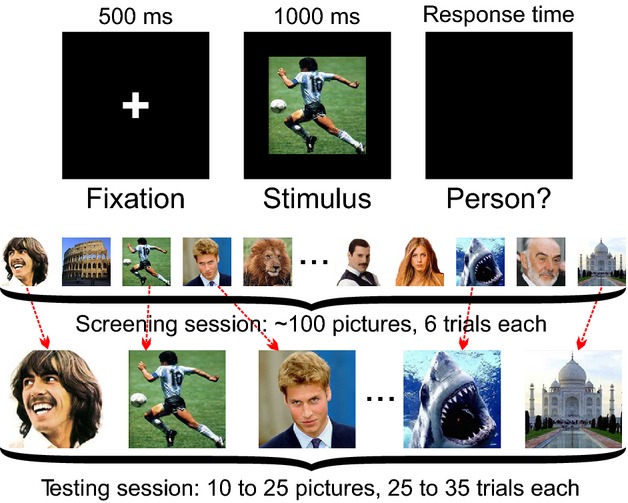
Example of screening and testing sessions. (Top) Each trial started with a fixation cross on the screen for 500 ms, followed by a picture displayed for 1000 ms. Next, the screen went black, and the patient had to press a key to respond whether or not there was a person in the picture. Finally, there was a random inter-trial interval between 600 and 800 ms. (Middle) A screening session is performed by showing a set of ∼100 pictures of animals, celebrities and landmarks, with each picture presented six times. (Bottom) The testing session is performed within a few hours after the screening session. In this case, a set of pictures eliciting responses in the screening session is shown again many more times to study the stability of the responses (see Fig.8).

In general, intracranial recordings have been helpful to improve our understanding in many topics in cognitive neuroscience (Mukamel & Fried, 2012). In this work we will focus on the behavior and function of ‘concept cells’ found in the human MTL (Quian Quiroga, 2012a). These neurons have very selective responses triggered by only a few stimuli. In order to find such stimuli, a simple visual task is used (Fig.2), in which the subject is sat facing a laptop computer where a set of about 100 stimuli are presented for 1 s, six times each in pseudorandom order (Quian Quiroga et al. 2005). To make sure subjects pay attention to the stimuli, after the picture presentation they have to respond whether or not there was a person in the picture by pressing different arrow keys. These ‘screening sessions’ typically last about half an hour, and they are conducted in order to determine which neurons are responsive and to which set of pictures. The set of pictures used includes items well familiar to the patient, like images of celebrities, landmarks, animals, and the patient’s relatives and friends. This set of pictures is actually tuned for each patient according to their preferences and background, as it has been shown that MTL neurons respond preferentially to personally relevant images (Viskontas et al. 2009).

Once it has been identified which picture/s triggers the firing of which neuron, different experimental paradigms are performed shortly after in order to test the firing patterns of these neurons under different tasks and conditions. The data shown in Figs6 were obtained this way by presenting many repetitions of the stimuli eliciting responses in the screening sessions. In the first study describing concept cells (Quian Quiroga et al. 2005), different pictures of the persons/objects eliciting responses in the screening sessions were shown to the subjects in the following sessions. This allowed establishing a very high degree of visual invariance of these responses, as the neurons tended to fire selectively to the different pictures of the same person and even to the person’s name written on the screen. A following study showed that such a degree of invariance extended to other sensory modalities, as these neurons also fired selectively when the name of the person eliciting a response (and not other names) was pronounced by a computer-synthetized voice (Quian Quiroga et al. 2009). Besides the interest of the results obtained from the screening sessions per se – to characterize the neurons’ selectivity (Waydo et al. 2006; Quian Quiroga et al. 2007), strength and latency of responses (Mormann et al. 2008; Quian Quiroga et al. 2009), preferential tuning (Viskontas et al. 2009; Mormann et al. 2011), correlation with LFP responses (Kraskov et al. 2007), repetition suppression effects (Pedreira et al. 2010), differential firing of pyramidal cells and interneurons (Ison et al. 2011), etc. – other experiments that explicitly used the knowledge of pictures triggering responses to study different properties of these neurons include a paradigm showing these pictures at the threshold of conscious perception (Quian Quiroga et al. 2008b), in a change blindness experiment (Reddy et al. 2006), another paradigm studying these neurons’ responses during free recall (Gelbard-Sagiv et al. 2008), another one assessing how these neurons rapidly encode new associations (Ison et al. 2010), and an experiment where the patients could voluntary control by internal thought which image from a pair (for which there were neurons responding) was displayed in a computer screen (Cerf et al. 2010).

**Figure 3:**
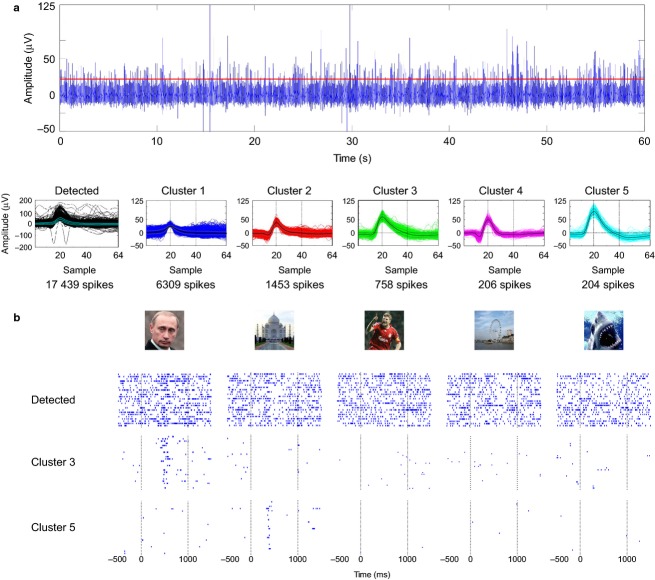
Single-unit responses. (a) Spike sorting. The top plot shows 60 s of the high-pass filtered recording (300–3000 Hz) from a microwire implanted in the left anterior hippocampus (the red solid line is the detection threshold). The bottom part shows all the detected spikes (left) and five clusters that were identified after sorting the spikes. (b) Raster plots associated to five pictures used during the session (first trial on top; time zero corresponds to stimulus onset). When all the detected spikes are considered (first row), there are no clear responses. However, the response is actually elicited by the single unit associated to cluster 3. Moreover, the raster plots for cluster 5 allow us to unravel a response to the picture of the Taj Mahal (that was not evident from the detected spikes). This cell fired selectively to this particular stimulus, remaining nearly silent to all the other stimuli.

**Figure 4:**
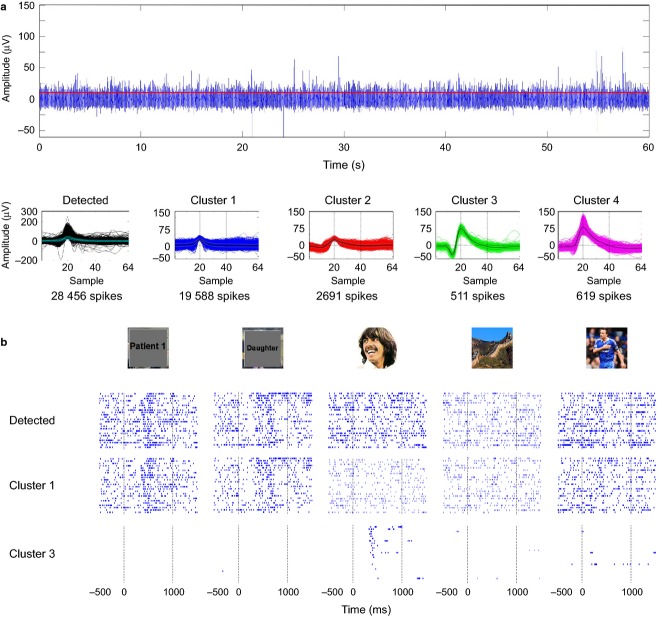
Multi and single unit responses. (a) Same conventions as in Fig.3. In this case, four clusters were identified after sorting the spikes recorded from a microwire implanted in the left anterior hippocampus. (b) Cluster 1 is associated to multi-unit activity showing responses to the pictures of the patient and his daughter (pictures covered for confidentiality issues). In addition, cluster 3 is an example of another silent neuron, in this case one responding selectively to the picture of George Harrison.

**Figure 5:**
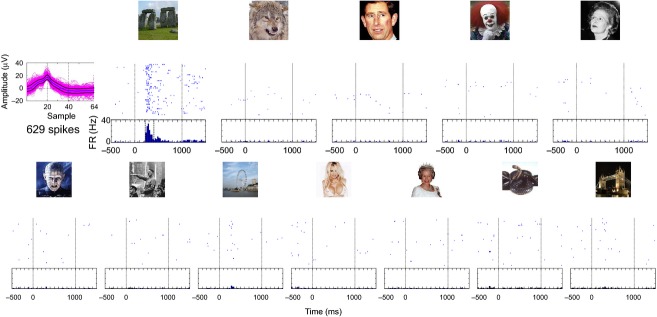
Selectivity of single-cell responses. Responses of a single unit in the left hippocampus during a testing session, where 12 pictures were presented 35 times each (19 min recording). The dashed vertical lines in the histogram denote onset and offset of the response based on the automatic response criterion. This ‘silent’ neuron (0.5 Hz on average during the session) shows a very selective response to the picture of Stonehenge (45% of the spikes took place in the trials associated to the Stonehenge picture, with half of them appearing between the onset and offset of the response).

**Figure 6:**
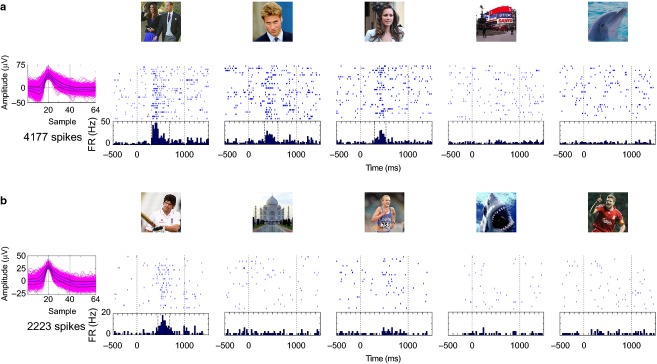
Stimulus relevance. (a) This unit in the left anterior hippocampus responded selectively to the pictures of Prince William, the Duchess of Cambridge (Kate Middleton) and both of them together, in a recording that took place 4 weeks before the ‘Royal Wedding’. (b) This unit in the left posterior hippocampus shows a selective response to the picture of Alastair Cook, a cricket player who was the ‘Player of the Ashes Series’ between England and Australia, which finished 2 months before the date of the recording.

## The importance of spike sorting

Action potentials (spikes) detected from implanted microwires might come from several neurons close enough to the electrode tip (Martinez et al. 2009; Quian Quiroga & Panzeri, 2009). The first step to process these recordings is to detect the spikes in the background noise (mainly generated by the activity of neurons further away), and to determine which spike corresponds to which neuron based on the spike shapes, a process called spike sorting (Quian Quiroga, 2012b). For the data presented here, spike detection and sorting was performed using Wave_Clus (Quian Quiroga et al. 2004). Briefly, the steps typically involved in spike detection and sorting are: (i) band-pass filtering the data, for example between 300 and 3000 Hz, using a zero-phase filter to avoid distortion in the spike waveforms (Quian Quiroga, 2009); (ii) computation of a detection threshold based on a robust estimate of the noise statistics; (iii) feature extraction of the spike shapes, in the case of Wave_Clus using the wavelet transform; and (iv) clustering of the waveforms to identify the firing of the different units.

The importance of spike sorting is illustrated with the example of Fig.3. Figure3a shows the result of the sorting of the spikes recorded by an electrode in the left hippocampus. Throughout the 16-min recording, a total of 17 439 spikes was detected. However, these spikes belong to different units. In particular, clusters 3 and 5 are associated to single units that account for only 4% and 1% of the detected spikes, respectively. As shown in Fig.3b, the unit in cluster 3 responds strongly to the picture of Vladimir Putin, with almost 20% of the spikes from this unit appearing between −500 and 1500 ms from the onset of this picture (representing 5% of the duration of the session). This response is more difficult to be visualized in the multiunit activity (i.e. just the detected spikes, without sorting). On the contrary, the response to the Taj Mahal from the unit in cluster 5, which also contains about 20% of the spikes from this unit, is not observed in the multiunit activity without the appropriate sorting. The fact that responses from sparsely firing or nearly silent neurons can be missed without optimal spike sorting (Quian Quiroga, 2012a; see also next section) is indeed a common finding with these recordings.

Another example of a ‘silent neuron’ is shown in Fig.4, where cluster 3, which accounts for 2% of the spikes detected in this channel, is associated to a single unit selectively responding to a picture of George Harrison (this response accounts for 15% of the spikes from this unit, representing 3% of the duration of the session). As before, it would have been impossible to identify this response from the detected spikes without optimal spike sorting. Figure4 shows also a multiunit cluster (cluster 1; i.e. a cluster where, due to low signal to noise ratio, it was not possible to separate the contribution of different units) that exhibits responses to a picture of the patient himself and to one of his daughter.

## Characterization of MTL single-cell responses

In the previous section we have illustrated some of the main properties of single-cell responses in the human MTL, particularly how they can selectively increase their firing from a very low baseline activity. As discussed above, the detection of these responses is challenging, first, due to the importance of optimal spike sorting and, second, due to the high selectivity of the responses and the need to identify which pictures should be used to trigger the neurons’ firing. The use of screening sessions together with optimal data processing of the data were indeed crucial to the finding of concept cells (Quian Quiroga et al. 2005). The most popular of these neurons, for example, responded to seven different pictures of Jennifer Aniston but not to 80 pictures of other celebrities, landmarks, animals, etc. The fact that these neurons fire to the concept represented by the stimulus and not to specific visual details, which could be common to a particular subset of pictures, is reinforced by the fact that the pictures of the same person used to test visual invariance included different backgrounds, poses, clothes, etc. More conclusive evidence of such conceptual representation is given by the fact that these neurons also fired to the written and spoken name of the person (or object) eliciting responses (Quian Quiroga et al. 2009).

### Selectivity

An important characteristic of these MTL neural responses is their high degree selectivity, with units responding on average only to 1–3% of the presented pictures (Quian Quiroga et al. 2007). In Fig.5, we show a characteristic example of a single unit in the hippocampus with a very selective response to a picture of Stonehenge. In fact, this sparsely firing neuron (0.5 Hz on average) fired 45% of the spikes recorded in the session in response to the picture of Stonehenge, while the rest occurred in response to the other 11 pictures or between trials.

Given the selectivity of these responses, the firing of these neurons was used to decode the identity of the person being shown, but it was in general not possible to decode which picture of the person was presented each time (Quian Quiroga et al. 2007). Therefore, these neurons give an explicit representation of the meaning of the stimuli (i.e. the neuron tells who the person being shown is).

Because these neurons fire to relatively very few stimuli, each stimulus is likely to be encoded by a relatively small neuronal assembly (Quian Quiroga et al. 2008a). In this ‘sparse coding’ view, the members of the assembly respond in an explicit manner to the specific concept they encode and typically remain silent for other objects. The extreme case would be a one-to-one correspondence between a single neuron and a particular concept, leading to the so-called ‘grandmother cells’ (Quian Quiroga et al. 2008a, 2013). But there are several reasons to reject the hypothesis of having one single cell per concept. Based on the total number of recorded neurons during a session, the number of stimuli presented and the number of responsive units, it has been estimated that out of about 10^9^ MTL neurons, < 10^6^ would be involved in representing a given concept (Waydo et al. 2006). Moreover, each MTL neuron was estimated to be involved in encoding between 50 and 150 distinct individuals or objects. As a matter of fact, these estimates should only be considered as upper bounds, given that: (i) the images used were personally relevant to the subject, thus increasing the likelihood of eliciting a response; (ii) the relatively low number of pictures used (about 100) limits the lowest selectivity value to 1%; and (iii) it is difficult to detect very selective neurons firing to even fewer pictures, which are likely not presented in the ∼30-min recording sessions.

With respect to the number of concepts encoded by these neurons, even though many responsive neurons fired to a single concept, it cannot be ruled out that they could have responded to other concepts if more pictures were shown. In fact, it is not uncommon to find units that do fire to more than one concept (Quian Quiroga et al. 2005, 2009). Such cases include responses to Jennifer Aniston and Lisa Kudrow (actresses of the series ‘Friends’), the Pisa and Eiffel towers (Quian Quiroga et al. 2005), Luke Skywalker and Yoda (Quian Quiroga et al. 2009), and Figs4 and 6a in this work. At the same time, neighboring neurons (e.g. units recorded in the same channel and separated by performing spike sorting) are typically involved in encoding completely unrelated concepts (see Fig.3; Quian Quiroga et al. 2009), which suggests a non-topographic organization.

It is also interesting to consider how such a sparse and conceptual representation may arise from the activity of upstream cortical areas. First of all, on the basis of their spike widths and firing rates it is in principle possible to classify neurons as interneurons or pyramidal cells. Using this classification, it was reported that MTL interneurons tend to fire to a larger number of stimuli, a finding that suggests a winner-takes-all mechanism that (by suppressing the firing of other neurons) may generate the very selective responses of pyramidal cells (Ison et al. 2011). Furthermore, by imposing a sparse representation in a detailed network model of visual processing, it was found that, without external supervision, the network learnt to discriminate persons or objects even if shown in different ways, as found in the human MTL (Waydo & Koch, 2008).

### Latency of MTL responses

From the examples we have already presented, it can be seen that the MTL responses have a very precise onset, typically between 200 and 600 ms after stimulus onset, with a mean latency of about 300 ms (Mormann et al. 2008). The timing of neural responses along the ventral visual pathway is given by direct feedforward projections (Thorpe & Fabre-Thorpe, 2001), culminating in responses at about 100 ms in the ITC in monkeys (Kreiman et al. 2006; see also table 1 in Mormann et al. 2008) and analogous structures in humans (Vanrullen & Thorpe, 2001; Liu et al. 2009). From ITC there are direct projections to the MTL (Saleem & Tanaka, 1996), but there is a gap of at least 100 ms between the responses in these two areas. A recent study (Rey et al. 2014) suggests that the timing of MTL single-cell responses may be given by LFP-evoked responses observed in this area. In particular, it was shown that single-neuron responses in the MTL were preceded by a global LFP deflection in the theta range (4–8 Hz), with the neurons’ firing locked to specific phases of the LFP (Rey et al. 2014). This theta phase locking was proposed to reflect a global activation that provides a temporal window for the processing of consciously perceived stimuli in the MTL. The timing mechanism given by the theta LFP responses may indeed be critical for synchronizing and combining multisensory information involving different processing times. The latency gap between ITC and MTL responses would then reflect the further processing of sensory stimuli (and perhaps the additional involvement of other areas, such as the prefrontal cortex) in order to create a unified conceptual representation that is used by the MTL for memory functions.

### Relationship with conscious perception

Studies using flash-suppression or very short presentations coupled with backward masking showed that the activity of these MTL neurons does not follow the retinal input but rather the subjective meaning of the perceived stimulus (Kreiman et al. 2002; Quian Quiroga et al. 2008b). In particular, it was shown that these neurons fired only when the subject recognized the stimulus eliciting responses and remained silent when it was not recognized, even if the visual stimuli were identical (i.e. the same picture at the same duration; Quian Quiroga et al. 2008b). In line with this evidence, the firing of these MTL neurons to the picture eliciting responses was higher when the subjects correctly identified a change in a change detection paradigm, compared with when they missed it (Reddy et al. 2006).

Given the vast amount of converging evidence supporting the role of the MTL in declarative memory processes but not in visual perception (Squire & Zola-Morgan, 1991; Eichenbaum, 2001; Squire et al. 2004; Moscovitch et al. 2005), it should be noted that this relationship between single-unit responses and stimulus recognition does not imply an involvement of the MTL in conscious visual perception. On the contrary, and as argued below, it likely reflects the subject’s awareness of the particular concepts being displayed for declarative, and particularly episodic, memory functions, like later remembering having seen a picture of Jennifer Aniston in the hospital ward (Quian Quiroga et al. 2008a).

### Internally generated responses

Another feature of these MTL responses is that they can be internally generated in the absence of the physical stimulus. When subjects imagined pictures that previously triggered the firing of a neuron (i.e. without the actual visual stimulus being shown), it was possible to see the same selective response as when viewing the picture (Kreiman et al. 2000b). Furthermore, Gelbard-Sagiv et al. (2008) found selective single-cell responses during the presentation of short video clips. Interestingly, they also found that when the subjects were asked to describe the various video clips that were presented (i.e. during free recall, in the absence of any sensory input), the same neurons that were active during stimulus encoding were reactivated during successful retrieval. The explicit representation of these MTL neurons was also exploited to achieve a voluntary modulation of the responses (Cerf et al. 2010): A target image was presented at the beginning of the trial, and from a 50 to 50% hybrid picture, the subjects could make a target picture clearer, fading out the other one, by voluntarily modifying the firing of the responsive MTL neurons. These results highlight the power of internal representation to override sensory input.

## Putative function of concept cells

As information progresses through the ventral visual pathway, it gets transformed from a detailed representation given by the receptive fields in the retina to a more abstract and conceptual representation in the MTL, where a large amount of details from low-level features are left behind. As discussed above, considering the overwhelming evidence linking the MTL to memory functions (Squire & Zola-Morgan, 1991; Eichenbaum, 2001; Squire et al. 2004; Moscovitch et al. 2005), it has been postulated that concept cells represent the meaning of the stimulus for declarative, and particularly episodic, memory functions (Quian Quiroga et al. 2008a; Quian Quiroga, 2012a). Representing the meaning of the stimulus involves a high degree of invariance, in the sense that these neurons respond to the particular concept being presented and disregard the varying details constituting the specific sensory representations. As also discussed above, these neurons do not act in isolation and a given concept is represented by the firing of a corresponding cell assembly. Sensory stimuli related to this concept, such as a picture or the written or spoken name of a person, would then activate different subsets of the assembly, which in turn activate the whole assembly via pattern completion (Quian Quiroga, 2012a).

A key mechanism related to episodic memories is the fast creation of new associations, like remembering meeting a person in a particular place. In this respect, it has been shown that MTL neurons can form new invariant responses and associations relatively quickly, for example, by responding to researchers performing experiments that were previously unknown to the patient (Quian Quiroga et al. 2009). Supporting this view of concept cells related to memory, patients with MTL lesions are impaired at accessing and combining contextual information and at providing detailed accounts of past experiences (Moscovitch et al. 2005) or even imagining new situations (Hassabis et al. 2007). Moreover, concept cells fired even in the absence of physical stimuli, triggered by internal thought or recall. A recent study in humans has also strengthened the link between MTL and memory function by showing that the phase locking between spiking and theta activity in this area during encoding predicted memory success (Rutishauser et al. 2010).

The high degree of visual invariance found in the human MTL has not been matched in other animals. In monkeys, the ITC shows a distributed code and limited robustness to basic image transformations (Logothetis & Sheinberg, 1996; Tanaka, 1996; Hung et al. 2005; Kreiman et al. 2006). Still, it is likely that, at least up to some degree, similar conceptual representations may exist in other species. Face-selective brain regions in the temporal lobe of monkeys, also known as face patches, have shown different tuning properties (Freiwald & Tsao, 2010). In particular, cells in the anterior medial patch show sparse, individual-specific, view-invariant response patterns. As this neural population approaches a view-invariant representation of facial identity, they serve as the closest experimental finding to concept cells in humans. In addition, concept cells share many features with place cells in the rodent hippocampus (Quian Quiroga, 2012a). Place cells increase their firing rate when the animal crosses a specific location in an environment (O’keefe & Nadel, 1978). They are selective, firing strongly to a particular place field, and they show fast learning, with its tuning being able to be formed within minutes (Wilson & McNaughton, 1993). They also show a non-topographic organization, in the sense that neighboring place fields do not correspond to nearby neurons (Redish et al. 2001). Moreover, as concept cells, place cells can fire even without visual information, as they maintain their tuning after the light is turned off, i.e. without visual information (Quirk et al. 1990). In line, it has been suggested that rather than just encoding a ‘cognitive map’, place cells may be part of a more general ‘memory space’ by encoding the common features that link different episodes, particularly linking episodes that occur at the same location (Eichenbaum et al. 1999; Eichenbaum, 2004).

## Stimulus relevance

Given the proposed role of concept cells in declarative memory, in the following we focus on two characteristics of these neurons that support this interpretation. First, if MTL neurons are involved in extracting the meaning of a stimulus for memory functions, it is likely that, due to the inherently subjective nature of such an operation, it would depend on the relevance and connotation that the stimulus has for the subject (Quian Quiroga, 2012a). In line, it has been shown that pictures of family members, including pictures of the patients themselves (see Fig.4b), and pictures of the researchers performing experiments with the patients, who are in close contact with them throughout their stay in hospital, are the most likely to elicit responses, followed by pictures of celebrities, and then by pictures of unfamiliar faces (Viskontas et al. 2009). In other words, we can infer that the more personally relevant the stimulus, the larger the number of neurons encoding it. This finding is consistent with the larger extent of episodic recollections and associations with other related concepts that personally relevant images generate.

Stimulus relevance is given by the subject’s own interest but also by the specific circumstances of what may be relevant at a particular time. To illustrate this, Fig.6a shows an example of a responsive unit in the anterior hippocampus that fired to the pictures of Prince William, the Duchess of Cambridge (Kate Middleton) and both of them together. Although the patient later stated being not particularly fond of matters related to the Royal family, the royal wedding took place about a month after the recording was performed and, being all over the media, this fact was therefore likely to be within the network of concepts and associations being salient for the subject at the time. We can also speculate that for the subject such an event might have later lost the relevance it had at the time, probably affecting the size of the cell assembly encoding these concepts, and the remembered associations and episodes linked to them. Another example comes from a unit in the posterior hippocampus (Fig.6b), which responded selectively to a picture of England’s cricket player Alastair Cook. This recording was performed 2 months after a series of test matches between England and Australia, in which Cook excelled and commanded the English team to victory.

The tendency of concept cells to preferentially encode items that are relevant at the time the experiments are performed is reinforced by other facts. For example, the researchers that performed experiments were completely unknown to the patients before the experiments took place, but they have a very frequent interaction with the patients while they are being monitored with intracranial recordings. Therefore, it is not surprising that their pictures and the ones of family members had the largest likelihood to elicit responses. This likelihood was followed by pictures of celebrities and finally by pictures of unknown faces (Viskontas et al. 2009). Naturally, family members are associated to remote (and/or recent) personal memories, while the experimenters are only associated to recent ones. This, in turn, raises the issue of whether MTL neurons are just involved in consolidation of new memories into cortex or whether they show a more stable representation even after consolidation, with responses decaying at a relatively slower rate given that episodes and associations related to specific concepts become less relevant and are forgotten with time. In the next section we will address this issue by studying variations of response strength with stimulus repetition.

## Response modulation by stimulus repetition

The ITC is the last purely visual area in the ventral visual pathway, and its neurons extract information about shapes, colors and even categories of objects (e.g. faces; Gross, 2008). An interesting phenomenon in monkey ITC is called ‘repetition suppression’: when the presentation of a novel stimulus is repeated several times, ITC neurons decrease the strength of their response (Li et al. 1993). Repetition suppression is stimulus specific, so these neurons are not just ‘novelty detectors’. However, the effect in ITC shows greater stimulus selectivity than the responses themselves (Sawamura et al. 2006), which might be due to a reflection of selectivity at the input rather than at the output level of a neuron. Repetition suppression is a common feature of neurons not just in ITC but also in medial temporal and prefrontal cortices (Ranganath & Rainer, 2003).

In humans, repetition suppression has been studied using non-invasive techniques (see Grill-Spector et al. 2006 for a review), such as fMRI (Sayres & Grill-Spector, 2006) and scalp EEG (Gruber & Müller, 2005). In particular, an fMRI study during a continuous recognition task with four presentations of test items showed that MTL structures exhibited a decrease in their activity for old vs. new items (Johnson et al. 2008). Moreover, it was also found that this effect can be graded, with continuing reductions after each successive presentation. However, repetition suppression in humans has been seen mostly at the category level (e.g. faces or scenes), failing to achieve the selective type of responses found in monkey ITC. One exception is given by a recent study with human single-cell recordings, in which the responses of several screening sessions (as the ones described in the Experimental paradigms section) were analyzed (Pedreira et al. 2010). In this study only the very first screening sessions were considered, so the specific pictures used were completely novel in the first trial. The activity of these neurons decayed with trial number, resembling the repetition suppression effect found in monkeys, and it was argued that this pattern of responses may reflect the decrease of relevant information to be stored into memory after each presentation (Pedreira et al. 2010).

The screening data used in the study of Pedreira and colleagues had only six presentations of the stimuli. Although a response modulation with stimulus repetition was evident, the stability of these representations over several trials could not be addressed, as it was hard to see a steady state in the response strength. Therefore, the question of whether the response would end up reaching a baseline level after a sufficient number of repetitions remained open. In order to study the stability of the representations by concept cells, we performed ‘testing sessions’, in which a subset of 10–25 pictures from the screening session (including all the ones that elicited a response) were used, but each of these images was shown 25–35 times in pseudorandom order (Fig.2, bottom). We studied recordings during 13 testing sessions in four patients (all right-handed, three male, 23–49 years old). From the 441 channels analyzed in this study, 37% showed spiking activity from 457 units (either multi- or single-unit).

After spikes were detected and sorted, it was necessary to quantitatively assess whether a particular unit responded to a particular picture. For this, we defined a responsiveness criterion that balanced the trade-off between false-positives and misses. According to our criterion, a responsive unit should fulfill the following conditions. First, in the response period (i.e. between 200 and 600 ms after stimulus onset) the unit should have a median number of spikes (across trials) larger than 1, to rule out neurons with too few spikes, and larger than *μ*_b _+ 4*σ*_b_, where *μ*_b_ and *σ*_b_ are the mean and standard deviation (across all stimuli) of the median number of spikes in the baseline period (between −600 and −200 ms). Second, it should have an average instantaneous firing rate (calculated by convolving the spike train with a Gaussian kernel) crossing over a threshold *T* for at least 100 ms (with the upwards and downwards crossings defining the spiking response onset and offset, respectively). The threshold *T* was set to *μ*_FR _+ 4*σ*_FR_, where *μ*_FR_ and *σ*_FR_ are the mean and standard deviation (across all stimuli) of the instantaneous firing rate during the baseline window (with a minimum at 5 Hz, for neurons with low baseline firing). Given that these MTL responses have a very well-defined onset (Mormann et al. 2008), this condition was introduced to avoid false-positive detections due to random increases spread over time. Third, to assure that the neuron’s response was consistent across trials, we compared the single-trial spike counts in the post-stimulus (200 ms, 600 ms) and baseline (−600 ms, −200 ms) windows with a paired sign test and required a significant difference with *P *< 0.01.

**Figure 7:**
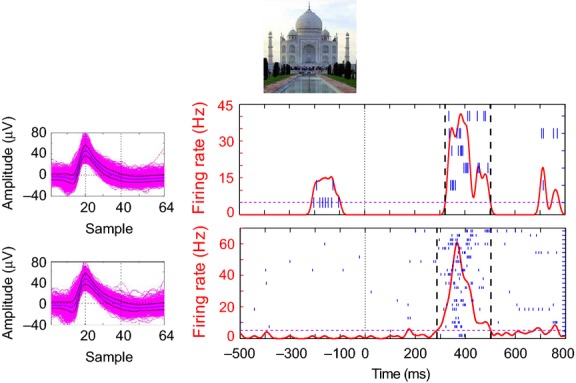
Response criterion. Example of a unit in the left hippocampus responsive to the picture of the Taj Mahal. The top panel shows the results from the first screening session. On the left, the waveforms of the 3520 spikes are superimposed. On the right, the raster plot associated to the picture of the Taj Mahal is shown (in blue). The red curve corresponds to the time profile of the mean firing rate (obtained by convolving the spike train with a Gaussian kernel with a value of *σ* = 10 ms), whereas the vertical dashed lines mark the crossing of a threshold (magenta line), and mark the onset and offset of the response. The bottom panel shows the equivalent results from (putatively) the same neuron (2112 spikes), but during the testing session (5 h after the first one).

Using this criterion, we identified a total of 72 responses (eight from amygdala and 64 from hippocampus), coming from 45 different units in 35 different channels. To illustrate the experimental and data processing approach detailed above, in Fig.7 we show the responses of a unit in the left hippocampus that selectively fired to the picture of the Taj Mahal. The top panel shows the raster plot for the screening session (six trials), and in the bottom panel the response of the same neuron obtained 5 h later during a following testing session, using 25 repetitions of a subset of 14 pictures. Both in the screening and the testing sessions we observe a similar response to the picture of the Taj Mahal.

**Figure 8:**
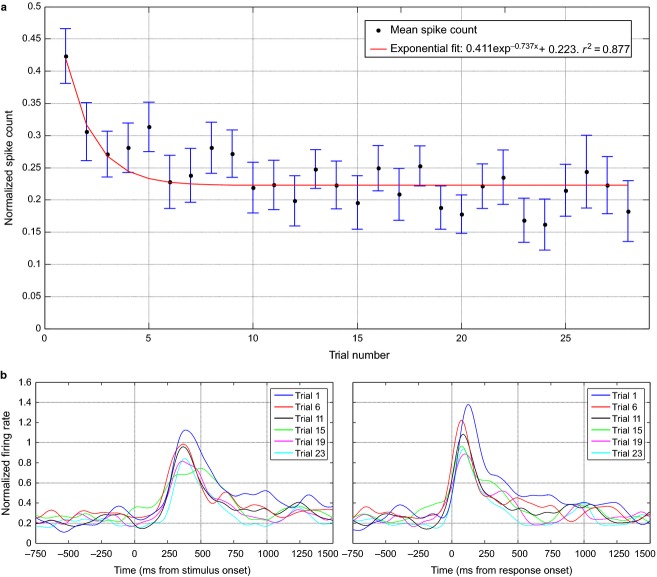
Response dynamics with stimulus repetition. (a) The figure shows the difference of the normalized spike count between the post-stimulus and baseline windows as a function of the trial number (number of times a particular stimulus was presented). The mean (dots) and SEM (error bars) were computed across the 72 responses found in the data set. The mean values were also fitted with an exponential model (solid line). (b) Time course of the mean normalized firing rate (across the 72 responses) for a group of selected trials. In the left panel, the time courses of each response for trial *i* are aligned to the stimulus onset before averaging, whereas in the right panel they are aligned to the response onset. For the computation of the instantaneous firing rate we used the Gaussian kernel with a value of *σ* = 50 ms.

If *D* is the duration of a response (time between onset and offset of the response), we defined a baseline window from max(−1000, −200 − 5*D) to −200, and a post-stimulus window between the response onset and min(onset + 5*D, 2000), where the times are measured in milliseconds from stimulus onset. The spike count on both windows was normalized for each response by the maximum (across trials) count in the post-stimulus window. As a first point in the analysis of these responses, to verify that repetition suppression effects were not due to varying levels of awareness, attention, etc. during the recording session, we checked that the normalized spike count in the baseline period showed no differences across trials (Kruskal–Wallis test, *P *= 0.6). Then, we computed the difference between the normalized spike count in post-stimulus and baseline windows (Fig.8a). When looking at the first six trials, we observe a repetition suppression effect similar to the one reported in previous studies with stimulus-selective neurons (Li et al. 1993; Pedreira et al. 2010). For example, the decay of the response is well fitted by an exponential function. However, the larger number of repetitions used here lets us unveil that the response has a clear non-zero steady state, which is reached after ∼10 trials (as in Sawamura et al. 2006). In other words, although after several repetitions the response strength was smaller than in the first trial, it remained well above baseline even after about 30 repetitions, with about 15 intervening stimuli on average. Although we cannot rule out a further decrease after many more repetitions, it seems that concept cells continue responding to a stimulus above baseline. This relatively stable representation resembles the findings with place cells in rodents, as it has been shown that, unless the environment is changed, place cells can maintain the same tuning properties for months (Thompson & Best, 1990).

To investigate the repetition suppression effect in more detail, we analyzed the time profile of the neural responses across trials. Figure8b shows the averaged normalized firing rate for a selection of trials at the different stages of the experimental sessions. A similar analysis was performed in Pedreira et al. (2010) and Li et al. (1993), but only using the first six repetitions. In line with these studies, we found a similar response onset for the different trials (Fig.8b, left), with responses starting to deviate from baseline at about 250 ms after stimulus onset. The right plot of Fig.8b shows the same results but aligning the instantaneous firing rate curves of each response to their corresponding response onsets. Confirming the findings of a previous study with a different dataset (Pedreira et al. 2010), we observe a decrease in the peak latency with trial number, which is related to a shortening of the response duration. There is also a large decay in peak amplitude, which was much less pronounced in Pedreira et al. (2010) as in that case only the first six trials were considered. Moreover, we observe no clear differences in the time profile of the responses after trial 15.

In summary, we showed that MTL neurons have a stable representation. In particular, the screening sessions used in Pedreira et al. (2010) were the very first experiments done with the patients, so the first trial corresponds to the very first presentation of each stimulus. Given the strong responses found from the first trial, we can infer that concept cells were involved in the encoding of the concept (which had not necessarily been active in the recent past) before the experiment took place. In fact, a non-stable, temporary representation of concepts would have implied a reassignment of these concepts to random sets of neurons after the first presentation, which would have taken at least a few trials to establish. Following these arguments, we can hypothesize that the stable representation by concept cells might last for as long as the concept remains relevant. The loss of relevance may then diminish the size of the cell assembly encoding the concept, and this may constitute a key neural mechanism of forgetting (Quian Quiroga, 2012a).

## Concluding remarks

In this work we have described the main procedures to obtain single-cell recordings from the human MTL. We have stressed the importance of using optimal experimental designs (with screening sessions) and data processing methodologies (particularly spike sorting) in order to identify the activity of sparsely firing neurons. The evidence reviewed above suggests that the activity of neurons in this area is related to the conscious processing of stimuli perceived by the subjects. The activation of a neuron associated to a certain concept is invariant to transformations of the stimuli (and even independent of the sensory modality), selective and explicit. In addition, concept cells tend to represent personally relevant concepts, as expected from their proposed role in episodic memory. Finally, we have presented a characterization of the stability with stimulus repetition of responses by concept cells, showing a decrease in response strength similar to the one described in high-level visual areas in monkeys. However, the response strength reached an asymptotic value larger than baseline firing, even after more than 20 presentations. Altogether, we postulate that the exploitation of data obtained from intracranial recordings (performed for clinical reasons) and, particularly, the further study of concept cells, can unveil key mechanisms implemented in the brain for the storage and retrieval of episodic memories.
